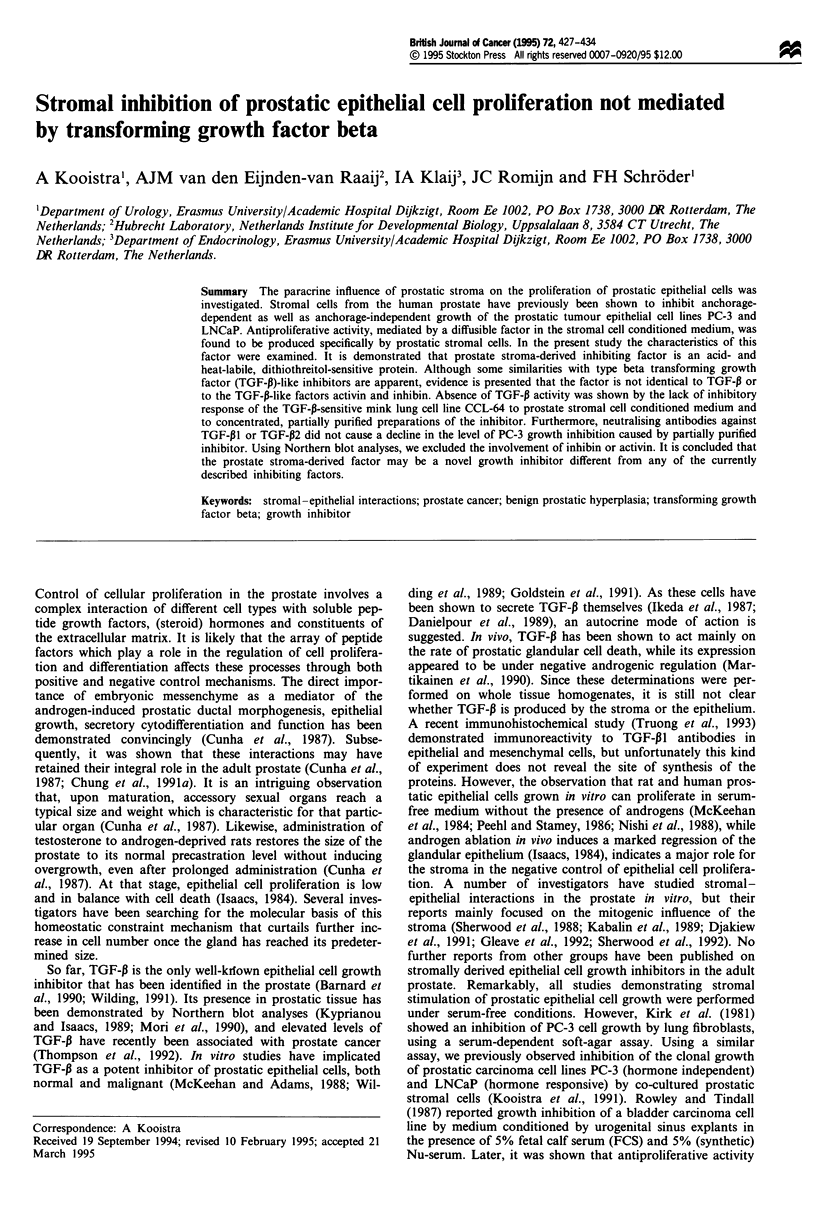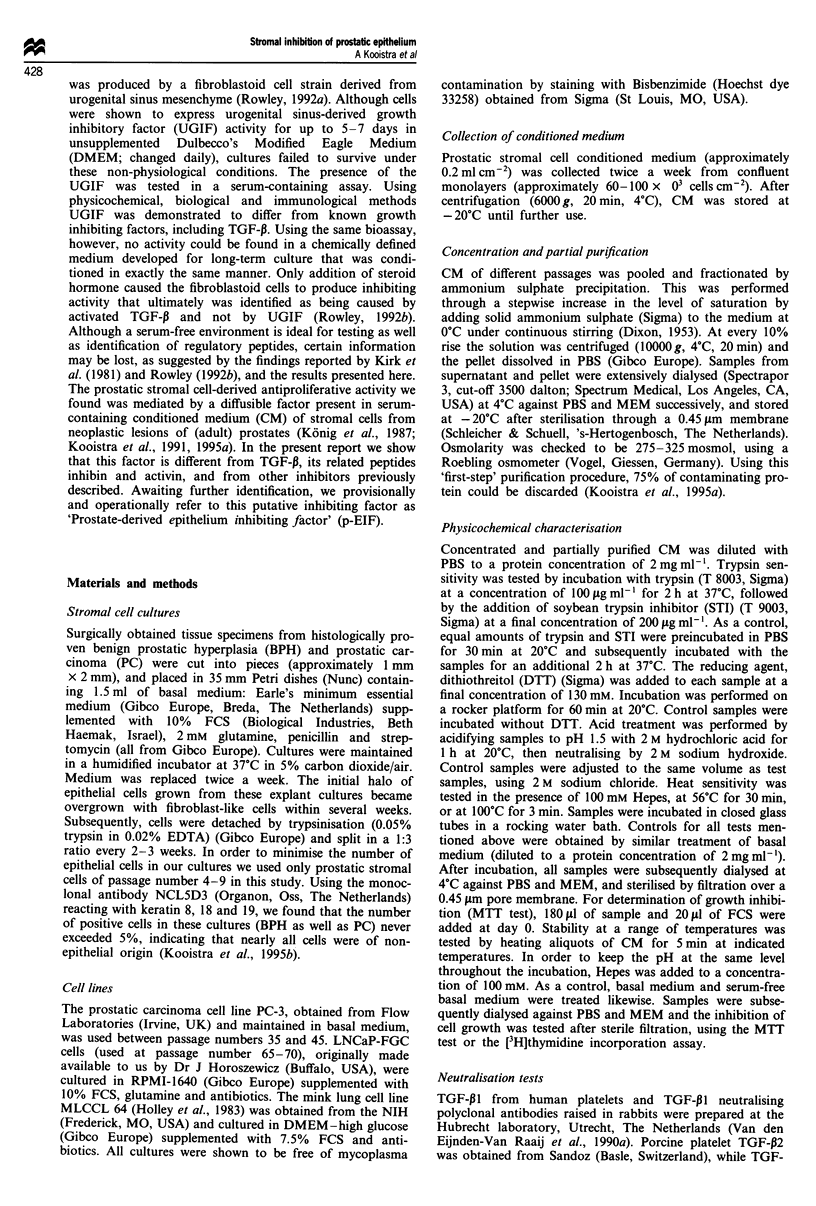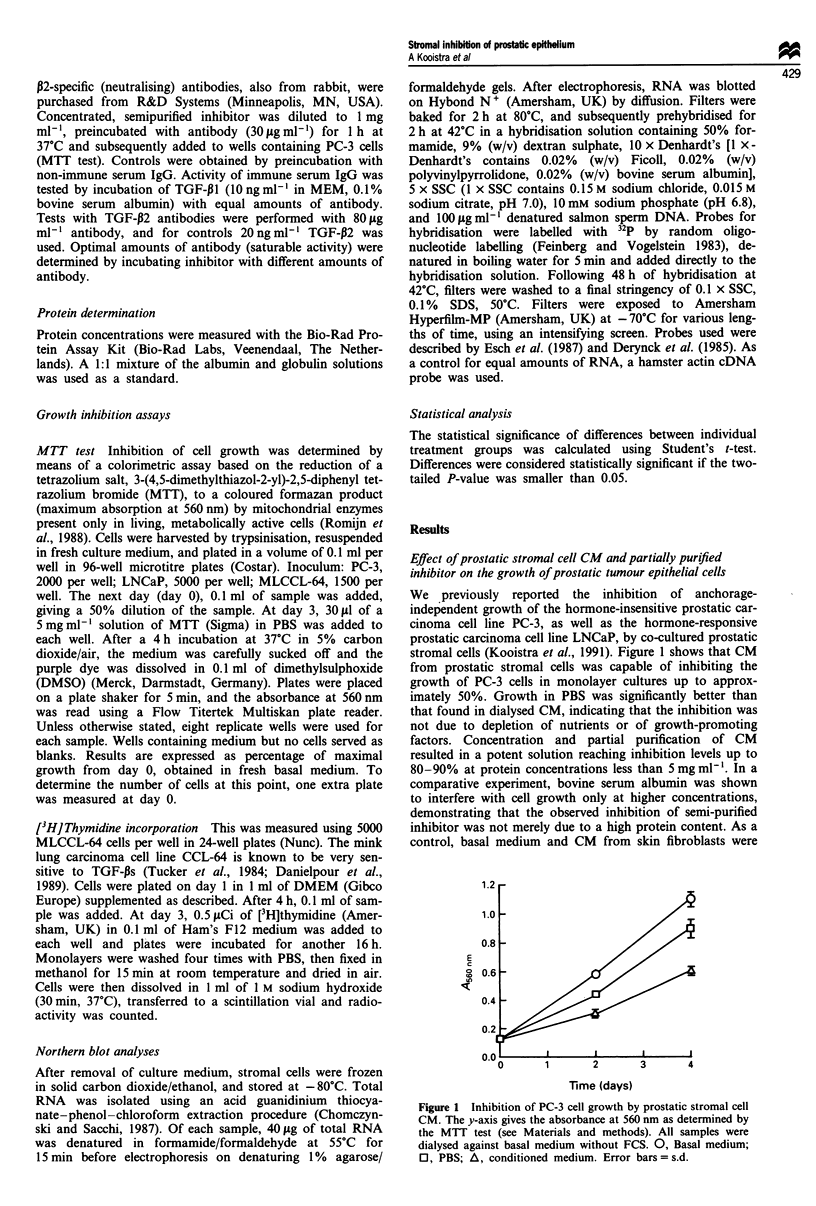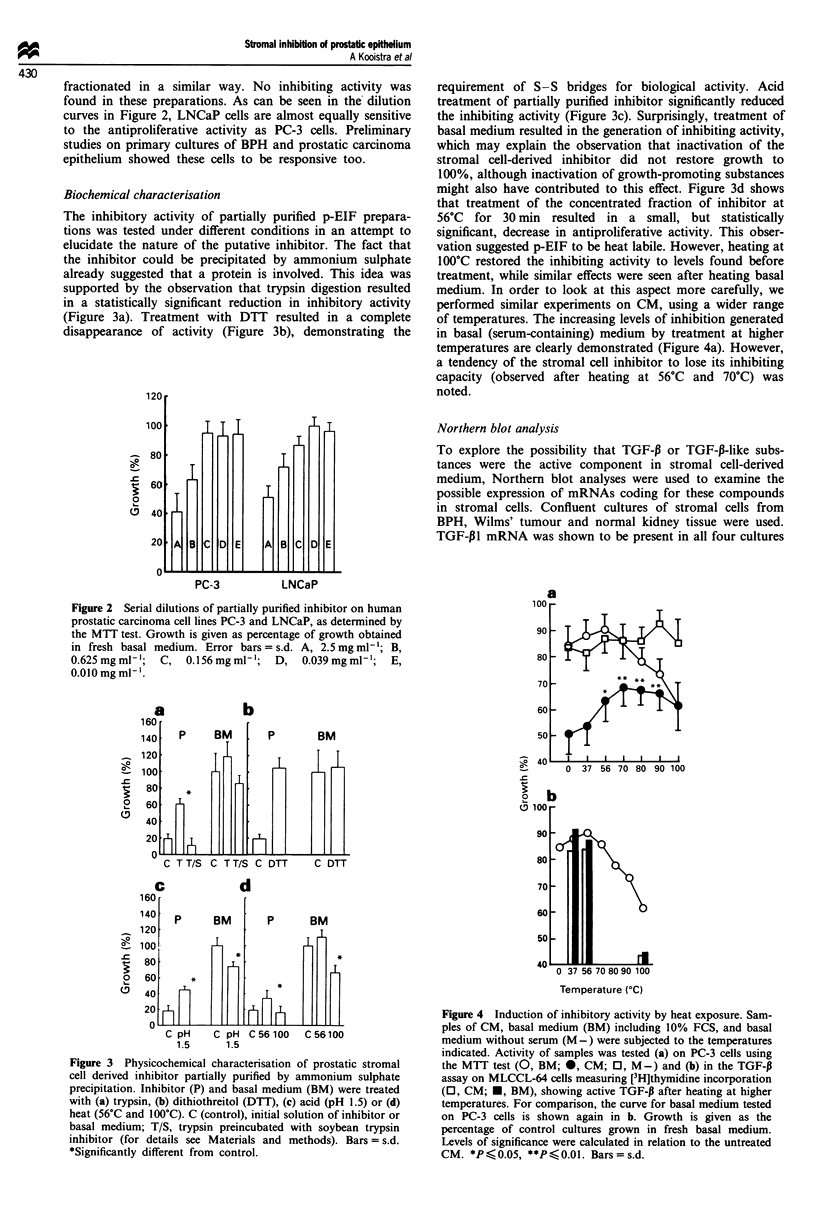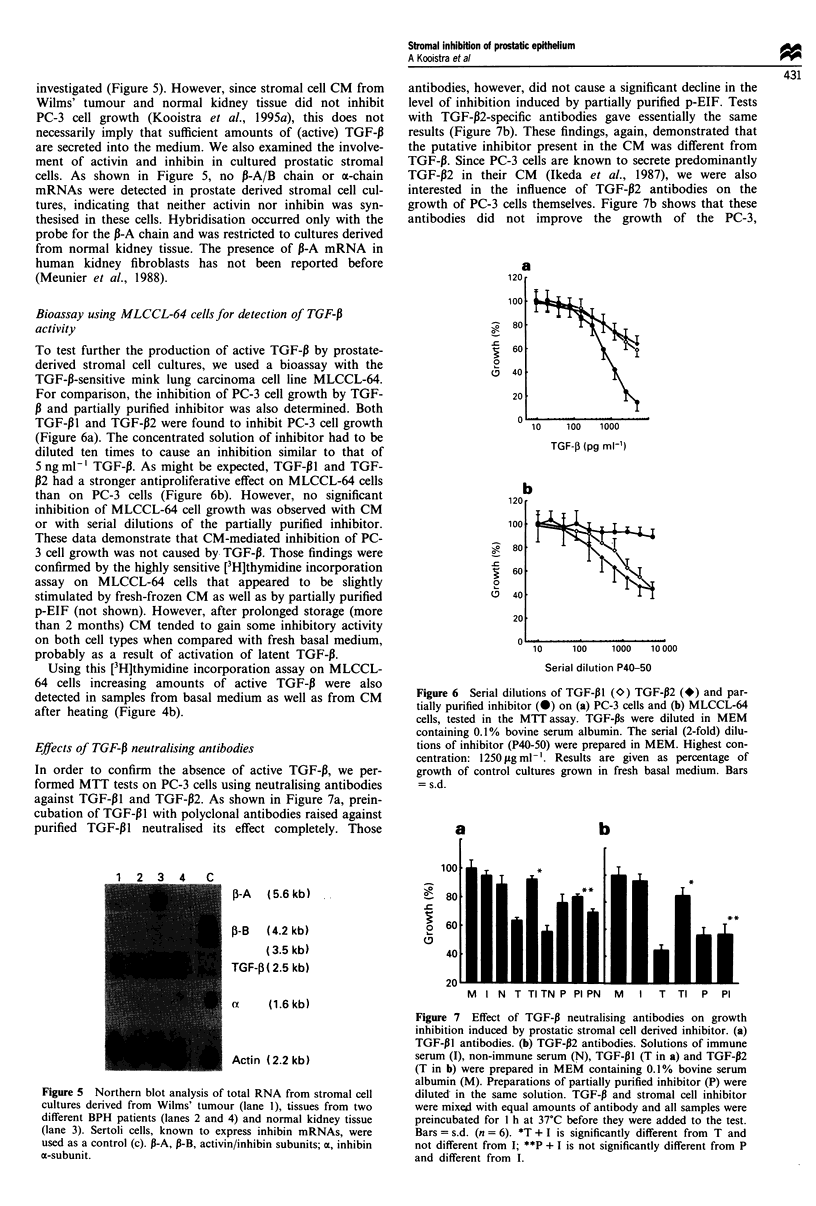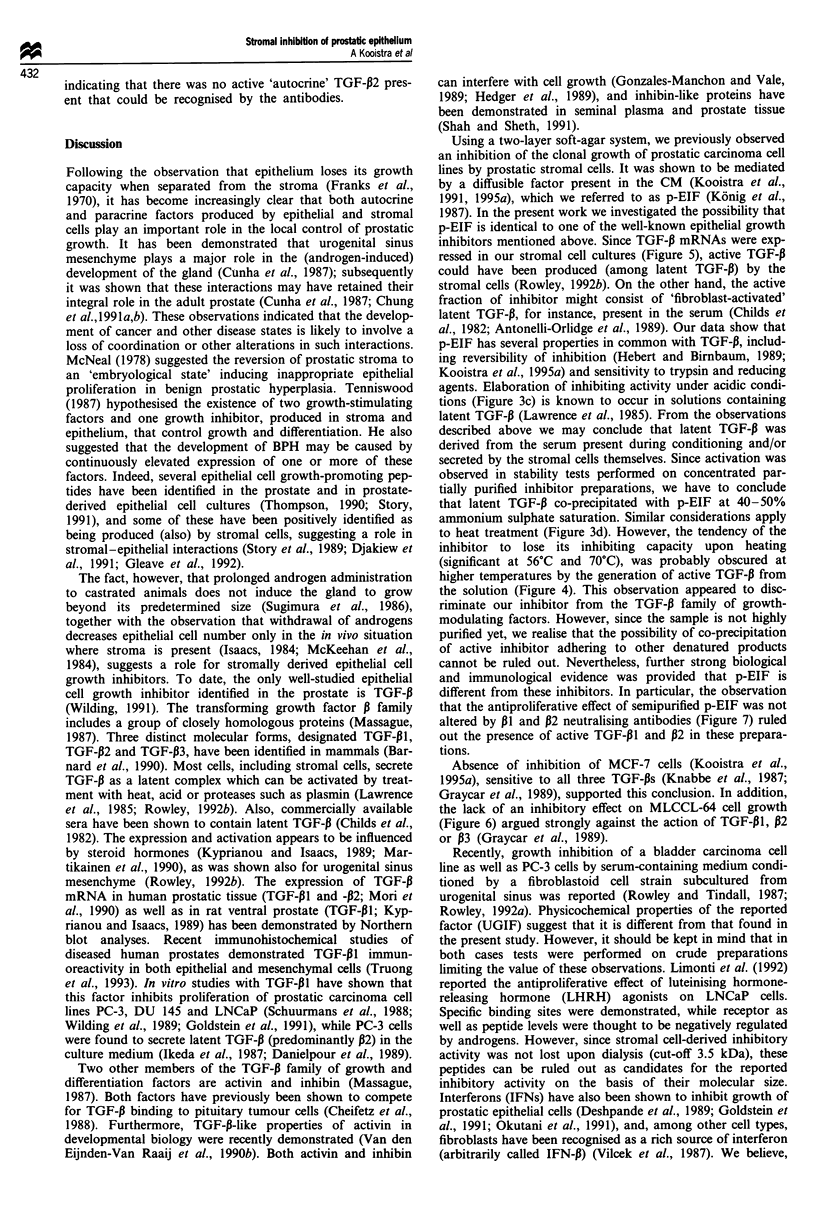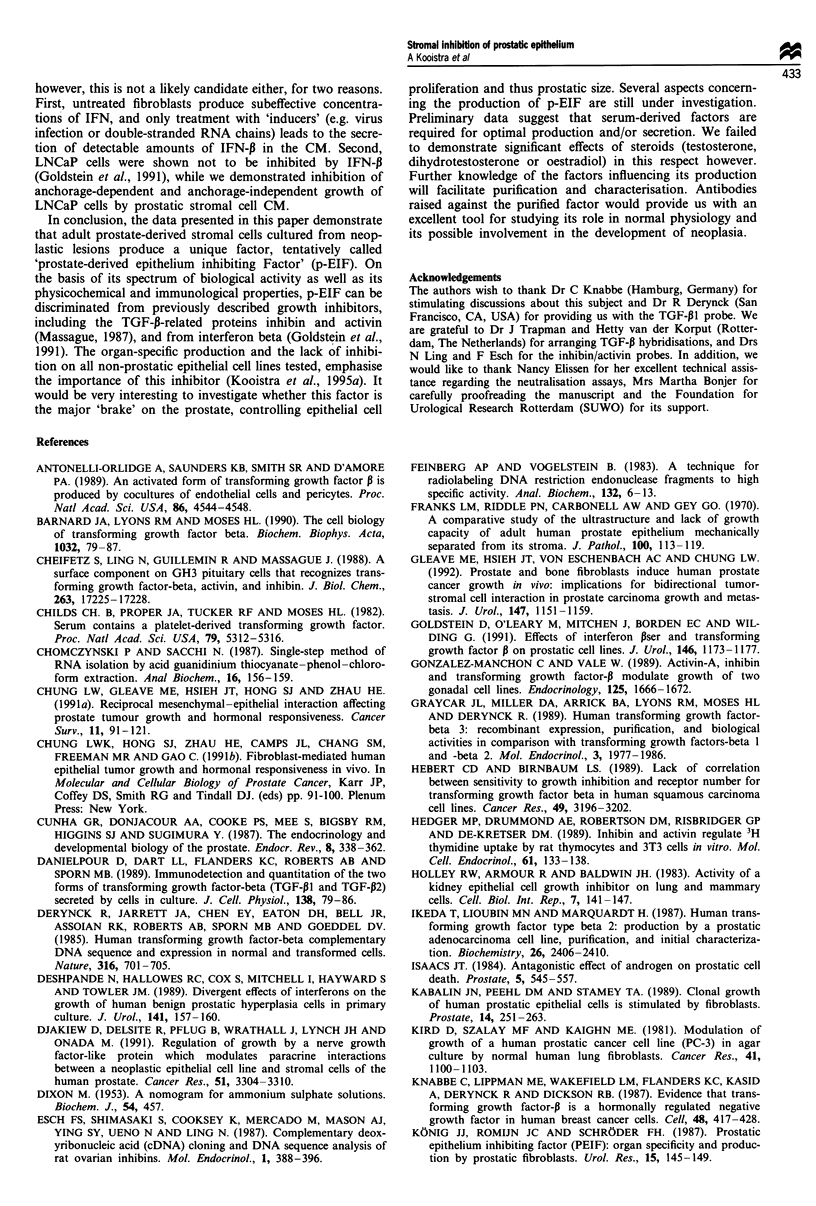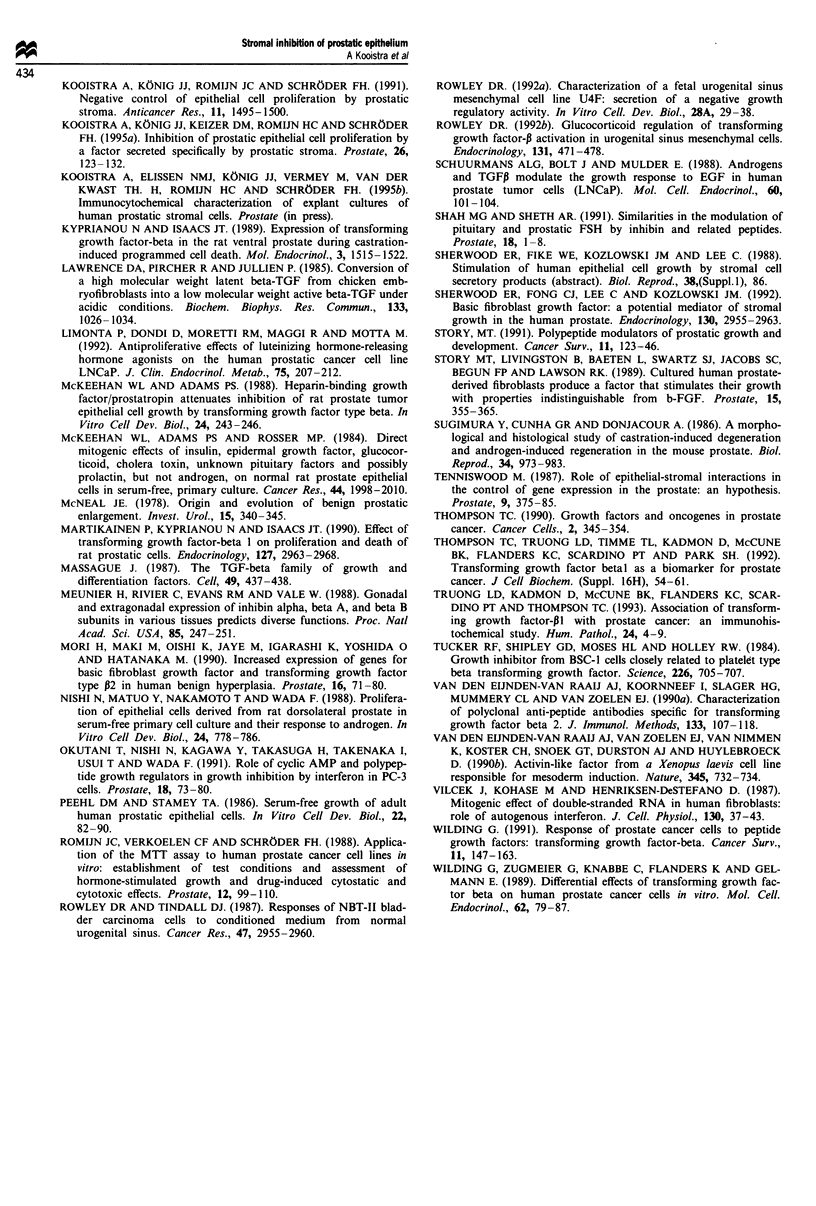# Stromal inhibition of prostatic epithelial cell proliferation not mediated by transforming growth factor beta.

**DOI:** 10.1038/bjc.1995.350

**Published:** 1995-08

**Authors:** A. Kooistra, A. J. van den Eijnden-van Raaij, I. A. Klaij, J. C. Romijn, F. H. Schröder

**Affiliations:** Department of Urology, Erasmus University/Academic Hospital Dijkzigt, Rotterdam, The Netherlands.

## Abstract

**Images:**


British Journal of Cancer (1995) 72, 427–434
© 1995 Stockton Press All rights reserved 0007-0920/95 $12.00

### Stromal inhibition of prostatic epithelial cell proliferation not mediated by transforming growth factor beta

A Kooistra[1], AJM van den Eijnden-van Raaij[2], IA Klaij[3], JC Romijn and FH Schröder[1]

[1]*Department of Urology, Erasmus University/Academic Hospital Dijkzigt, Room Ee 1002, PO Box 1738, 3000 DR Rotterdam, The Netherlands;* [2]*Hubrecht Laboratory, Netherlands Institute for Developmental Biology, Uppsalalaan 8, 3584 CT Utrecht, The Netherlands;* [3]*Department of Endocrinology, Erasmus University/Academic Hospital Dijkzigt, Room Ee 1002, PO Box 1738, 3000 DR Rotterdam, The Netherlands.*

**Summary** The paracrine influence of prostatic stroma on the proliferation of prostatic epithelial cells was investigated. Stromal cells from the human prostate have previously been shown to inhibit anchorage-dependent as well as anchorage-independent growth of the prostatic tumour epithelial cell lines PC-3 and LNCaP. Antiproliferative activity, mediated by a diffusible factor in the stromal cell conditioned medium, was found to be produced specifically by prostatic stromal cells. In the present study the characteristics of this factor were examined. It is demonstrated that prostate stroma-derived inhibiting factor is an acid- and heat-labile, dithiothreitol-sensitive protein. Although some similarities with type beta transforming growth factor (TGF-β)-like inhibitors are apparent, evidence is presented that the factor is not identical to TGF-β or to the TGF-β-like factors activin and inhibin. Absence of TGF-β activity was shown by the lack of inhibitory response of the TGF-β-sensitive mink lung cell line CCL-64 to prostate stromal cell conditioned medium and to concentrated, partially purified preparations of the inhibitor. Furthermore, neutralising antibodies against TGF-β1 or TGF-β2 did not cause a decline in the level of PC-3 growth inhibition caused by partially purified inhibitor. Using Northern blot analyses, we excluded the involvement of inhibin or activin. It is concluded that the prostate stroma-derived factor may be a novel growth inhibitor different from any of the currently described inhibiting factors.

**Keywords:** stromal–epithelial interactions; prostate cancer; benign prostatic hyperplasia; transforming growth factor beta; growth inhibitor

Control of cellular proliferation in the prostate involves a complex interaction of different cell types with soluble peptide growth factors, (steroid) hormones and constituents of the extracellular matrix. It is likely that the array of peptide factors which play a role in the regulation of cell proliferation and differentiation affects these processes through both positive and negative control mechanisms. The direct importance of embryonic messenchyme as a mediator of the androgen-induced prostatic ductal morphogenesis, epithelial growth, secretory cytodifferentiation and function has been demonstrated convincingly (Cunha *et al.*, 1987). Subsequently, it was shown that these interactions may have retained their integral role in the adult prostate (Cunha *et al.*, 1987; Chung *et al.*, 1991*a*). It is an intriguing observation that, upon maturation, accessory sexual organs reach a typical size and weight which is characteristic for that particular organ (Cunha *et al.*, 1987). Likewise, administration of testosterone to androgen-deprived rats restores the size of the prostate to its normal precastration level without inducing overgrowth, even after prolonged administration (Cunha *et al.*, 1987). At that stage, epithelial cell proliferation is low and in balance with cell death (Isaacs, 1984). Several investigators have been searching for the molecular basis of this homeostatic constraint mechanism that curtails further increase in cell number once the gland has reached its predetermined size.

So far, TGF-β is the only well-known epithelial cell growth inhibitor that has been identified in the prostate (Barnard *et al.*, 1990; Wilding, 1991). Its presence in prostatic tissue has been demonstrated by Northern blot analyses (Kyprianou and Isaacs, 1989; Mori *et al.*, 1990), and elevated levels of TGF-β have recently been associated with prostate cancer (Thompson *et al.*, 1992). *In vitro* studies have implicated TGF-β as a potent inhibitor of prostatic epithelial cells, both normal and malignant (McKeehan and Adams, 1988; Wilding *et al.*, 1989; Goldstein *et al.*, 1991). As these cells have been shown to secrete TGF-β themselves (Ikeda *et al.*, 1987; Danielpour *et al.*, 1989), an autocrine mode of action is suggested. *In vivo*, TGF-β has been shown to act mainly on the rate of prostatic glandular cell death, while its expression appeared to be under negative androgenic regulation (Martikainen *et al.*, 1990). Since these determinations were performed on whole tissue homogenates, it is still not clear whether TGF-β is produced by the stroma or the epithelium. A recent immunohistochemical study (Truong *et al.*, 1993) demonstrated immunoreactivity to TGF-β1 antibodies in epithelial and mesenchymal cells, but unfortunately this kind of experiment does not reveal the site of synthesis of the proteins. However, the observation that rat and human prostatic epithelial cells grown *in vitro* can proliferate in serum-free medium without the presence of androgens (McKeehan *et al.*, 1984; Peehl and Stamey, 1986; Nishi *et al.*, 1988), while androgen ablation *in vivo* induces a marked regression of the glandular epithelium (Isaacs, 1984), indicates a major role for the stroma in the negative control of epithelial cell proliferation. A number of investigators have studied stromal–epithelial interactions in the prostate *in vitro*, but their reports mainly focused on the mitogenic influence of the stroma (Sherwood *et al.*, 1988; Kabalin *et al.*, 1989; Djakiew *et al.*, 1991; Gleave *et al.*, 1992; Sherwood *et al.*, 1992). No further reports from other groups have been published on stromally derived epithelial cell growth inhibitors in the adult prostate. Remarkably, all studies demonstrating stromal stimulation of prostatic epithelial cell growth were performed under serum-free conditions. However, Kirk *et al.* (1981) showed an inhibition of PC-3 cell growth by lung fibroblasts, using a serum-dependent soft-agar assay. Using a similar assay, we previously observed inhibition of the clonal growth of prostatic carcinoma cell lines PC-3 (hormone independent) and LNCaP (hormone responsive) by co-cultured prostatic stromal cells (Kooistra *et al.*, 1991). Rowley and Tindall (1987) reported growth inhibition of a bladder carcinoma cell line by medium conditioned by urogenital sinus explants in the presence of 5% fetal calf serum (FCS) and 5% (synthetic) Nu-serum. Later, it was shown that antiproliferative activity

Correspondence: A Kooistra
Received 19 September 1994; revised 10 February 1995; accepted 21 March 1995

was produced by a fibroblastoid cell strain derived from urogenital sinus mesenchyme (Rowley, 1992*a*). Although cells were shown to express urogenital sinus-derived growth inhibitory factor (UGIF) activity for up to 5–7 days in unsupplemented Dulbecco's Modified Eagle Medium (DMEM; changed daily), cultures failed to survive under these non-physiological conditions. The presence of the UGIF was tested in a serum-containing assay. Using physicochemical, biological and immunological methods UGIF was demonstrated to differ from known growth inhibiting factors, including TGF-$\beta$. Using the same bioassay, however, no activity could be found in a chemically defined medium developed for long-term culture that was conditioned in exactly the same manner. Only addition of steroid hormone caused the fibroblastoid cells to produce inhibiting activity that ultimately was identified as being caused by activated TGF-$\beta$ and not by UGIF (Rowley, 1992*b*). Although a serum-free environment is ideal for testing as well as identification of regulatory peptides, certain information may be lost, as suggested by the findings reported by Kirk *et al.* (1981) and Rowley (1992*b*), and the results presented here. The prostatic stromal cell-derived antiproliferative activity we found was mediated by a diffusible factor present in serum-containing conditioned medium (CM) of stromal cells from neoplastic lesions of (adult) prostates (König *et al.*, 1987; Kooistra *et al.*, 1991, 1995*a*). In the present report we show that this factor is different from TGF-$\beta$, its related peptides inhibin and activin, and from other inhibitors previously described. Awaiting further identification, we provisionally and operationally refer to this putative inhibiting factor as 'Prostate-derived *e*pithelium *i*nhibiting *f*actor' (p-EIF).

### Materials and methods

### *Stromal cell cultures*

Surgically obtained tissue specimens from histologically proven benign prostatic hyperplasia (BPH) and prostatic carcinoma (PC) were cut into pieces (approximately 1 mm × 2 mm), and placed in 35 mm Petri dishes (Nunc) containing 1.5 ml of basal medium: Earle's minimum essential medium (Gibco Europe, Breda, The Netherlands) supplemented with 10% FCS (Biological Industries, Beth Haemak, Israel), 2 mM glutamine, penicillin and streptomycin (all from Gibco Europe). Cultures were maintained in a humidified incubator at 37°C in 5% carbon dioxide/air. Medium was replaced twice a week. The initial halo of epithelial cells grown from these explant cultures became overgrown with fibroblast-like cells within several weeks. Subsequently, cells were detached by trypsinisation (0.05% trypsin in 0.02% EDTA) (Gibco Europe) and split in a 1:3 ratio every 2–3 weeks. In order to minimise the number of epithelial cells in our cultures we used only prostatic stromal cells of passage number 4–9 in this study. Using the monoclonal antibody NCL5D3 (Organon, Oss, The Netherlands) reacting with keratin 8, 18 and 19, we found that the number of positive cells in these cultures (BPH as well as PC) never exceeded 5%, indicating that nearly all cells were of non-epithelial origin (Kooistra *et al.*, 1995*b*).

### *Cell lines*

The prostatic carcinoma cell line PC-3, obtained from Flow Laboratories (Irvine, UK) and maintained in basal medium, was used between passage numbers 35 and 45. LNCaP-FGC cells (used at passage number 65–70), originally made available to us by Dr J Horoszewicz (Buffalo, USA), were cultured in RPMI-1640 (Gibco Europe) supplemented with 10% FCS, glutamine and antibiotics. The mink lung cell line MLCCL 64 (Holley *et al.*, 1983) was obtained from the NIH (Frederick, MO, USA) and cultured in DMEM–high glucose (Gibco Europe) supplemented with 7.5% FCS and antibiotics. All cultures were shown to be free of mycoplasma contamination by staining with Bisbenzimide (Hoechst dye 33258) obtained from Sigma (St Louis, MO, USA).

### *Collection of conditioned medium*

Prostatic stromal cell conditioned medium (approximately 0.2 ml cm$^{-2}$) was collected twice a week from confluent monolayers (approximately 60–100 × 0$^3$ cells cm$^{-2}$). After centrifugation (6000 *g*, 20 min, 4°C), CM was stored at −20°C until further use.

### *Concentration and partial purification*

CM of different passages was pooled and fractionated by ammonium sulphate precipitation. This was performed through a stepwise increase in the level of saturation by adding solid ammonium sulphate (Sigma) to the medium at 0°C under continuous stirring (Dixon, 1953). At every 10% rise the solution was centrifuged (10000 *g*, 4°C, 20 min) and the pellet dissolved in PBS (Gibco Europe). Samples from supernatant and pellet were extensively dialysed (Spectrapor 3, cut-off 3500 dalton; Spectrum Medical, Los Angeles, CA, USA) at 4°C against PBS and MEM successively, and stored at −20°C after sterilisation through a 0.45 $\mu$m membrane (Schleicher & Schuell, 's-Hertogenbosch, The Netherlands). Osmolarity was checked to be 275–325 mosmol, using a Roebling osmometer (Vogel, Giessen, Germany). Using this 'first-step' purification procedure, 75% of contaminating protein could be discarded (Kooistra *et al.*, 1995*a*).

### *Physicochemical characterisation*

Concentrated and partially purified CM was diluted with PBS to a protein concentration of 2 mg ml$^{-1}$. Trypsin sensitivity was tested by incubation with trypsin (T 8003, Sigma) at a concentration of 100 $\mu$g ml$^{-1}$ for 2 h at 37°C, followed by the addition of soybean trypsin inhibitor (STI) (T 9003, Sigma) at a final concentration of 200 $\mu$g ml$^{-1}$. As a control, equal amounts of trypsin and STI were preincubated in PBS for 30 min at 20°C and subsequently incubated with the samples for an additional 2 h at 37°C. The reducing agent, dithiothreitol (DTT) (Sigma) was added to each sample at a final concentration of 130 mM. Incubation was performed on a rocker platform for 60 min at 20°C. Control samples were incubated without DTT. Acid treatment was performed by acidifying samples to pH 1.5 with 2 M hydrochloric acid for 1 h at 20°C, then neutralising by 2 M sodium hydroxide. Control samples were adjusted to the same volume as test samples, using 2 M sodium chloride. Heat sensitivity was tested in the presence of 100 mM Hepes, at 56°C for 30 min, or at 100°C for 3 min. Samples were incubated in closed glass tubes in a rocking water bath. Controls for all tests mentioned above were obtained by similar treatment of basal medium (diluted to a protein concentration of 2 mg ml$^{-1}$). After incubation, all samples were subsequently dialysed at 4°C against PBS and MEM, and sterilised by filtration over a 0.45 $\mu$m pore membrane. For determination of growth inhibition (MTT test), 180 $\mu$l of sample and 20 $\mu$l of FCS were added at day 0. Stability at a range of temperatures was tested by heating aliquots of CM for 5 min at indicated temperatures. In order to keep the pH at the same level throughout the incubation, Hepes was added to a concentration of 100 mM. As a control, basal medium and serum-free basal medium were treated likewise. Samples were subsequently dialysed against PBS and MEM and the inhibition of cell growth was tested after sterile filtration, using the MTT test or the [$^3$H]thymidine incorporation assay.

### *Neutralisation tests*

TGF-$\beta$1 from human platelets and TGF-$\beta$1 neutralising polyclonal antibodies raised in rabbits were prepared at the Hubrecht laboratory, Utrecht, The Netherlands (Van den Eijnden-Van Raaij *et al.*, 1990*a*). Porcine platelet TGF-$\beta$2 was obtained from Sandoz (Basle, Switzerland), while TGF-

$\beta$2-specific (neutralising) antibodies, also from rabbit, were purchased from R&D Systems (Minneapolis, MN, USA). Concentrated, semipurified inhibitor was diluted to 1 mg $ml^{-1}$, preincubated with antibody (30 $\mu g\ ml^{-1}$) for 1 h at 37°C and subsequently added to wells containing PC-3 cells (MTT test). Controls were obtained by preincubation with non-immune serum IgG. Activity of immune serum IgG was tested by incubation of TGF-$\beta$1 (10 ng $ml^{-1}$ in MEM, 0.1% bovine serum albumin) with equal amounts of antibody. Tests with TGF-$\beta$2 antibodies were performed with 80 $\mu g\ ml^{-1}$ antibody, and for controls 20 ng $ml^{-1}$ TGF-$\beta$2 was used. Optimal amounts of antibody (saturable activity) were determined by incubating inhibitor with different amounts of antibody.

*Protein determination*

Protein concentrations were measured with the Bio-Rad Protein Assay Kit (Bio-Rad Labs, Veenendaal, The Netherlands). A 1:1 mixture of the albumin and globulin solutions was used as a standard.

*Growth inhibition assays*

*MTT test* Inhibition of cell growth was determined by means of a colorimetric assay based on the reduction of a tetrazolium salt, 3-(4,5-dimethylthiazol-2-yl)-2,5-diphenyl tetrazolium bromide (MTT), to a coloured formazan product (maximum absorption at 560 nm) by mitochondrial enzymes present only in living, metabolically active cells (Romijn *et al.*, 1988). Cells were harvested by trypsinisation, resuspended in fresh culture medium, and plated in a volume of 0.1 ml per well in 96-well microtitre plates (Costar). Inoculum: PC-3, 2000 per well; LNCaP, 5000 per well; MLCCL-64, 1500 per well. The next day (day 0), 0.1 ml of sample was added, giving a 50% dilution of the sample. At day 3, 30 $\mu l$ of a 5 mg $ml^{-1}$ solution of MTT (Sigma) in PBS was added to each well. After a 4 h incubation at 37°C in 5% carbon dioxide/air, the medium was carefully sucked off and the purple dye was dissolved in 0.1 ml of dimethylsulphoxide (DMSO) (Merck, Darmstadt, Germany). Plates were placed on a plate shaker for 5 min, and the absorbance at 560 nm was read using a Flow Titertek Multiskan plate reader. Unless otherwise stated, eight replicate wells were used for each sample. Wells containing medium but no cells served as blanks. Results are expressed as percentage of maximal growth from day 0, obtained in fresh basal medium. To determine the number of cells at this point, one extra plate was measured at day 0.

*[$^3$H]Thymidine incorporation* This was measured using 5000 MLCCL-64 cells per well in 24-well plates (Nunc). The mink lung carcinoma cell line CCL-64 is known to be very sensitive to TGF-$\beta$s (Tucker *et al.*, 1984; Danielpour *et al.*, 1989). Cells were plated on day 1 in 1 ml of DMEM (Gibco Europe) supplemented as described. After 4 h, 0.1 ml of sample was added. At day 3, 0.5 $\mu Ci$ of [$^3$H]thymidine (Amersham, UK) in 0.1 ml of Ham's F12 medium was added to each well and plates were incubated for another 16 h. Monolayers were washed four times with PBS, then fixed in methanol for 15 min at room temperature and dried in air. Cells were then dissolved in 1 ml of 1 M sodium hydroxide (30 min, 37°C), transferred to a scintillation vial and radioactivity was counted.

*Northern blot analyses*

After removal of culture medium, stromal cells were frozen in solid carbon dioxide/ethanol, and stored at −80°C. Total RNA was isolated using an acid guanidinium thiocyanate–phenol–chloroform extraction procedure (Chomczynski and Sacchi, 1987). Of each sample, 40 $\mu g$ of total RNA was denatured in formamide/formaldehyde at 55°C for 15 min before electrophoresis on denaturing 1% agarose/formaldehyde gels. After electrophoresis, RNA was blotted on Hybond N$^+$ (Amersham, UK) by diffusion. Filters were baked for 2 h at 80°C, and subsequently prehybridised for 2 h at 42°C in a hybridisation solution containing 50% formamide, 9% (w/v) dextran sulphate, 10 × Denhardt's [1 × Denhardt's contains 0.02% (w/v) Ficoll, 0.02% (w/v) polyvinylpyrrolidone, 0.02% (w/v) bovine serum albumin], 5 × SSC (1 × SSC contains 0.15 M sodium chloride, 0.015 M sodium citrate, pH 7.0), 10 mM sodium phosphate (pH 6.8), and 100 $\mu g\ ml^{-1}$ denatured salmon sperm DNA. Probes for hybridisation were labelled with $^{32}$P by random oligonucleotide labelling (Feinberg and Vogelstein 1983), denatured in boiling water for 5 min and added directly to the hybridisation solution. Following 48 h of hybridisation at 42°C, filters were washed to a final stringency of 0.1 × SSC, 0.1% SDS, 50°C. Filters were exposed to Amersham Hyperfilm-MP (Amersham, UK) at −70°C for various lengths of time, using an intensifying screen. Probes used were described by Esch *et al.* (1987) and Derynck *et al.* (1985). As a control for equal amounts of RNA, a hamster actin cDNA probe was used.

*Statistical analysis*

The statistical significance of differences between individual treatment groups was calculated using Student's *t*-test. Differences were considered statistically significant if the two-tailed *P*-value was smaller than 0.05.

### Results

*Effect of prostatic stromal cell CM and partially purified inhibitor on the growth of prostatic tumour epithelial cells*

We previously reported the inhibition of anchorage-independent growth of the hormone-insensitive prostatic carcinoma cell line PC-3, as well as the hormone-responsive prostatic carcinoma cell line LNCaP, by co-cultured prostatic stromal cells (Kooistra *et al.*, 1991). Figure 1 shows that CM from prostatic stromal cells was capable of inhibiting the growth of PC-3 cells in monolayer cultures up to approximately 50%. Growth in PBS was significantly better than that found in dialysed CM, indicating that the inhibition was not due to depletion of nutrients or of growth-promoting factors. Concentration and partial purification of CM resulted in a potent solution reaching inhibition levels up to 80–90% at protein concentrations less than 5 mg $ml^{-1}$. In a comparative experiment, bovine serum albumin was shown to interfere with cell growth only at higher concentrations, demonstrating that the observed inhibition of semi-purified inhibitor was not merely due to a high protein content. As a control, basal medium and CM from skin fibroblasts were

**Figure 1** Inhibition of PC-3 cell growth by prostatic stromal cell CM. The *y*-axis gives the absorbance at 560 nm as determined by the MTT test (see Materials and methods). All samples were dialysed against basal medium without FCS. O, Basal medium; □, PBS; Δ, conditioned medium. Error bars = s.d.

fractionated in a similar way. No inhibiting activity was found in these preparations. As can be seen in the dilution curves in Figure 2, LNCaP cells are almost equally sensitive to the antiproliferative activity as PC-3 cells. Preliminary studies on primary cultures of BPH and prostatic carcinoma epithelium showed these cells to be responsive too.

*Biochemical characterisation*

The inhibitory activity of partially purified p-EIF preparations was tested under different conditions in an attempt to elucidate the nature of the putative inhibitor. The fact that the inhibitor could be precipitated by ammonium sulphate already suggested that a protein is involved. This idea was supported by the observation that trypsin digestion resulted in a statistically significant reduction in inhibitory activity (Figure 3a). Treatment with DTT resulted in a complete disappearance of activity (Figure 3b), demonstrating the requirement of S–S bridges for biological activity. Acid treatment of partially purified inhibitor significantly reduced the inhibiting activity (Figure 3c). Surprisingly, treatment of basal medium resulted in the generation of inhibiting activity, which may explain the observation that inactivation of the stromal cell-derived inhibitor did not restore growth to 100%, although inactivation of growth-promoting substances might also have contributed to this effect. Figure 3d shows that treatment of the concentrated fraction of inhibitor at 56°C for 30 min resulted in a small, but statistically significant, decrease in antiproliferative activity. This observation suggested p-EIF to be heat labile. However, heating at 100°C restored the inhibiting activity to levels found before treatment, while similar effects were seen after heating basal medium. In order to look at this aspect more carefully, we performed similar experiments on CM, using a wider range of temperatures. The increasing levels of inhibition generated in basal (serum-containing) medium by treatment at higher temperatures are clearly demonstrated (Figure 4a). However, a tendency of the stromal cell inhibitor to lose its inhibiting capacity (observed after heating at 56°C and 70°C) was noted.

*Northern blot analysis*

To explore the possibility that TGF-$\beta$ or TGF-$\beta$-like substances were the active component in stromal cell-derived medium, Northern blot analyses were used to examine the possible expression of mRNAs coding for these compounds in stromal cells. Confluent cultures of stromal cells from BPH, Wilms' tumour and normal kidney tissue were used. TGF-$\beta$1 mRNA was shown to be present in all four cultures

**Figure 2** Serial dilutions of partially purified inhibitor on human prostatic carcinoma cell lines PC-3 and LNCaP, as determined by the MTT test. Growth is given as percentage of growth obtained in fresh basal medium. Error bars = s.d. A, 2.5 mg ml$^{-1}$; B, 0.625 mg ml$^{-1}$; C, 0.156 mg ml$^{-1}$; D, 0.039 mg ml$^{-1}$; E, 0.010 mg ml$^{-1}$.

**Figure 3** Physicochemical characterisation of prostatic stromal cell derived inhibitor partially purified by ammonium sulphate precipitation. Inhibitor (P) and basal medium (BM) were treated with (**a**) trypsin, (**b**) dithiothreitol (DTT), (**c**) acid (pH 1.5) or (**d**) heat (56°C and 100°C). C (control), initial solution of inhibitor or basal medium; T/S, trypsin preincubated with soybean trypsin inhibitor (for details see Materials and methods). Bars = s.d. *Significantly different from control.

**Figure 4** Induction of inhibitory activity by heat exposure. Samples of CM, basal medium (BM) including 10% FCS, and basal medium without serum (M–) were subjected to the temperatures indicated. Activity of samples was tested (**a**) on PC-3 cells using the MTT test (O, BM; ●, CM; □, M–) and (**b**) in the TGF-$\beta$ assay on MLCCL-64 cells measuring [$^3$H]thymidine incorporation (□, CM; ■, BM), showing active TGF-$\beta$ after heating at higher temperatures. For comparison, the curve for basal medium tested on PC-3 cells is shown again in **b**. Growth is given as the percentage of control cultures grown in fresh basal medium. Levels of significance were calculated in relation to the untreated CM. *$P \leq 0.05$, **$P \leq 0.01$. Bars = s.d.

investigated (Figure 5). However, since stromal cell CM from Wilms' tumour and normal kidney tissue did not inhibit PC-3 cell growth (Kooistra *et al.*, 1995*a*), this does not necessarily imply that sufficient amounts of (active) TGF-$\beta$ are secreted into the medium. We also examined the involvement of activin and inhibin in cultured prostatic stromal cells. As shown in Figure 5, no $\beta$-A/B chain or $\alpha$-chain mRNAs were detected in prostate derived stromal cell cultures, indicating that neither activin nor inhibin was synthesised in these cells. Hybridisation occurred only with the probe for the $\beta$-A chain and was restricted to cultures derived from normal kidney tissue. The presence of $\beta$-A mRNA in human kidney fibroblasts has not been reported before (Meunier *et al.*, 1988).

### *Bioassay using MLCCL-64 cells for detection of TGF-β activity*

To test further the production of active TGF-$\beta$ by prostate-derived stromal cell cultures, we used a bioassay with the TGF-$\beta$-sensitive mink lung carcinoma cell line MLCCL-64. For comparison, the inhibition of PC-3 cell growth by TGF-$\beta$ and partially purified inhibitor was also determined. Both TGF-$\beta$1 and TGF-$\beta$2 were found to inhibit PC-3 cell growth (Figure 6a). The concentrated solution of inhibitor had to be diluted ten times to cause an inhibition similar to that of 5 ng ml$^{-1}$ TGF-$\beta$. As might be expected, TGF-$\beta$1 and TGF-$\beta$2 had a stronger antiproliferative effect on MLCCL-64 cells than on PC-3 cells (Figure 6b). However, no significant inhibition of MLCCL-64 cell growth was observed with CM or with serial dilutions of the partially purified inhibitor. These data demonstrate that CM-mediated inhibition of PC-3 cell growth was not caused by TGF-$\beta$. Those findings were confirmed by the highly sensitive [$^3$H]thymidine incorporation assay on MLCCL-64 cells that appeared to be slightly stimulated by fresh-frozen CM as well as by partially purified p-EIF (not shown). However, after prolonged storage (more than 2 months) CM tended to gain some inhibitory activity on both cell types when compared with fresh basal medium, probably as a result of activation of latent TGF-$\beta$.

Using this [$^3$H]thymidine incorporation assay on MLCCL-64 cells increasing amounts of active TGF-$\beta$ were also detected in samples from basal medium as well as from CM after heating (Figure 4b).

### *Effects of TGF-β neutralising antibodies*

In order to confirm the absence of active TGF-$\beta$, we performed MTT tests on PC-3 cells using neutralising antibodies against TGF-$\beta$1 and TGF-$\beta$2. As shown in Figure 7a, preincubation of TGF-$\beta$1 with polyclonal antibodies raised against purified TGF-$\beta$1 neutralised its effect completely. Those antibodies, however, did not cause a significant decline in the level of inhibition induced by partially purified p-EIF. Tests with TGF-$\beta$2-specific antibodies gave essentially the same results (Figure 7b). These findings, again, demonstrated that the putative inhibitor present in the CM was different from TGF-$\beta$. Since PC-3 cells are known to secrete predominantly TGF-$\beta$2 in their CM (Ikeda *et al.*, 1987), we were also interested in the influence of TGF-$\beta$2 antibodies on the growth of PC-3 cells themselves. Figure 7b shows that these antibodies did not improve the growth of the PC-3,

**Figure 6** Serial dilutions of TGF-$\beta$1 (◇) TGF-$\beta$2 (◆) and partially purified inhibitor (●) on (**a**) PC-3 cells and (**b**) MLCCL-64 cells, tested in the MTT assay. TGF-$\beta$s were diluted in MEM containing 0.1% bovine serum albumin. The serial (2-fold) dilutions of inhibitor (P40-50) were prepared in MEM. Highest concentration: 1250 $\mu$g ml$^{-1}$. Results are given as percentage of growth of control cultures grown in fresh basal medium. Bars = s.d.

**Figure 5** Northern blot analysis of total RNA from stromal cell cultures derived from Wilms' tumour (lane 1), tissues from two different BPH patients (lanes 2 and 4) and normal kidney tissue (lane 3). Sertoli cells, known to express inhibin mRNAs, were used as a control (c). $\beta$-A, $\beta$-B, activin/inhibin subunits; $\alpha$, inhibin $\alpha$-subunit.

**Figure 7** Effect of TGF-$\beta$ neutralising antibodies on growth inhibition induced by prostatic stromal cell derived inhibitor. (**a**) TGF-$\beta$1 antibodies. (**b**) TGF-$\beta$2 antibodies. Solutions of immune serum (I), non-immune serum (N), TGF-$\beta$1 (T in **a**) and TGF-$\beta$2 (T in **b**) were prepared in MEM containing 0.1% bovine serum albumin (M). Preparations of partially purified inhibitor (P) were diluted in the same solution. TGF-$\beta$ and stromal cell inhibitor were mixed with equal amounts of antibody and all samples were preincubated for 1 h at 37°C before they were added to the test. Bars = s.d. ($n = 6$). *T + I is significantly different from T and not different from I; **P + I is not significantly different from P and different from I.

indicating that there was no active 'autocrine' TGF-β2 present that could be recognised by the antibodies.

### Discussion

Following the observation that epithelium loses its growth capacity when separated from the stroma (Franks *et al.*, 1970), it has become increasingly clear that both autocrine and paracrine factors produced by epithelial and stromal cells play an important role in the local control of prostatic growth. It has been demonstrated that urogenital sinus mesenchyme plays a major role in the (androgen-induced) development of the gland (Cunha *et al.*, 1987); subsequently it was shown that these interactions may have retained their integral role in the adult prostate (Cunha *et al.*, 1987; Chung *et al.*,1991*a*,*b*). These observations indicated that the development of cancer and other disease states is likely to involve a loss of coordination or other alterations in such interactions. McNeal (1978) suggested the reversion of prostatic stroma to an 'embryological state' inducing inappropriate epithelial proliferation in benign prostatic hyperplasia. Tenniswood (1987) hypothesised the existence of two growth-stimulating factors and one growth inhibitor, produced in stroma and epithelium, that control growth and differentiation. He also suggested that the development of BPH may be caused by continuously elevated expression of one or more of these factors. Indeed, several epithelial cell growth-promoting peptides have been identified in the prostate and in prostate-derived epithelial cell cultures (Thompson, 1990; Story, 1991), and some of these have been positively identified as being produced (also) by stromal cells, suggesting a role in stromal–epithelial interactions (Story *et al.*, 1989; Djakiew *et al.*, 1991; Gleave *et al.*, 1992).

The fact, however, that prolonged androgen administration to castrated animals does not induce the gland to grow beyond its predetermined size (Sugimura *et al.*, 1986), together with the observation that withdrawal of androgens decreases epithelial cell number only in the *in vivo* situation where stroma is present (Isaacs, 1984; McKeehan *et al.*, 1984), suggests a role for stromally derived epithelial cell growth inhibitors. To date, the only well-studied epithelial cell growth inhibitor identified in the prostate is TGF-β (Wilding, 1991). The transforming growth factor β family includes a group of closely homologous proteins (Massague, 1987). Three distinct molecular forms, designated TGF-β1, TGF-β2 and TGF-β3, have been identified in mammals (Barnard *et al.*, 1990). Most cells, including stromal cells, secrete TGF-β as a latent complex which can be activated by treatment with heat, acid or proteases such as plasmin (Lawrence *et al.*, 1985; Rowley, 1992*b*). Also, commercially available sera have been shown to contain latent TGF-β (Childs *et al.*, 1982). The expression and activation appears to be influenced by steroid hormones (Kyprianou and Isaacs, 1989; Martikainen *et al.*, 1990), as was shown also for urogenital sinus mesenchyme (Rowley, 1992*b*). The expression of TGF-β mRNA in human prostatic tissue (TGF-β1 and -β2; Mori *et al.*, 1990) as well as in rat ventral prostate (TGF-β1; Kyprianou and Isaacs, 1989) has been demonstrated by Northern blot analyses. Recent immunohistochemical studies of diseased human prostates demonstrated TGF-β1 immunoreactivity in both epithelial and mesenchymal cells (Truong *et al.*, 1993). *In vitro* studies with TGF-β1 have shown that this factor inhibits proliferation of prostatic carcinoma cell lines PC-3, DU 145 and LNCaP (Schuurmans *et al.*, 1988; Wilding *et al.*, 1989; Goldstein *et al.*, 1991), while PC-3 cells were found to secrete latent TGF-β (predominantly β2) in the culture medium (Ikeda *et al.*, 1987; Danielpour *et al.*, 1989).

Two other members of the TGF-β family of growth and differentiation factors are activin and inhibin (Massague, 1987). Both factors have previously been shown to compete for TGF-β binding to pituitary tumour cells (Cheifetz *et al.*, 1988). Furthermore, TGF-β-like properties of activin in developmental biology were recently demonstrated (Van den Eijnden-Van Raaij *et al.*, 1990*b*). Both activin and inhibin can interfere with cell growth (Gonzales-Manchon and Vale, 1989; Hedger *et al.*, 1989), and inhibin-like proteins have been demonstrated in seminal plasma and prostate tissue (Shah and Sheth, 1991).

Using a two-layer soft-agar system, we previously observed an inhibition of the clonal growth of prostatic carcinoma cell lines by prostatic stromal cells. It was shown to be mediated by a diffusible factor present in the CM (Kooistra *et al.*, 1991, 1995*a*), which we referred to as p-EIF (König *et al.*, 1987). In the present work we investigated the possibility that p-EIF is identical to one of the well-known epithelial growth inhibitors mentioned above. Since TGF-β mRNAs were expressed in our stromal cell cultures (Figure 5), active TGF-β could have been produced (among latent TGF-β) by the stromal cells (Rowley, 1992*b*). On the other hand, the active fraction of inhibitor might consist of 'fibroblast-activated' latent TGF-β, for instance, present in the serum (Childs *et al.*, 1982; Antonelli-Orlidge *et al.*, 1989). Our data show that p-EIF has several properties in common with TGF-β, including reversibility of inhibition (Hebert and Birnbaum, 1989; Kooistra *et al.*, 1995*a*) and sensitivity to trypsin and reducing agents. Elaboration of inhibiting activity under acidic conditions (Figure 3c) is known to occur in solutions containing latent TGF-β (Lawrence *et al.*, 1985). From the observations described above we may conclude that latent TGF-β was derived from the serum present during conditioning and/or secreted by the stromal cells themselves. Since activation was observed in stability tests performed on concentrated partially purified inhibitor preparations, we have to conclude that latent TGF-β co-precipitated with p-EIF at 40–50% ammonium sulphate saturation. Similar considerations apply to heat treatment (Figure 3d). However, the tendency of the inhibitor to lose its inhibiting capacity upon heating (significant at 56°C and 70°C), was probably obscured at higher temperatures by the generation of active TGF-β from the solution (Figure 4). This observation appeared to discriminate our inhibitor from the TGF-β family of growth-modulating factors. However, since the sample is not highly purified yet, we realise that the possibility of co-precipitation of active inhibitor adhering to other denatured products cannot be ruled out. Nevertheless, further strong biological and immunological evidence was provided that p-EIF is different from these inhibitors. In particular, the observation that the antiproliferative effect of semipurified p-EIF was not altered by β1 and β2 neutralising antibodies (Figure 7) ruled out the presence of active TGF-β1 and β2 in these preparations.

Absence of inhibition of MCF-7 cells (Kooistra *et al.*, 1995*a*), sensitive to all three TGF-βs (Knabbe *et al.*, 1987; Graycar *et al.*, 1989), supported this conclusion. In addition, the lack of an inhibitory effect on MLCCL-64 cell growth (Figure 6) argued strongly against the action of TGF-β1, β2 or β3 (Graycar *et al.*, 1989).

Recently, growth inhibition of a bladder carcinoma cell line as well as PC-3 cells by serum-containing medium conditioned by a fibroblastoid cell strain subcultured from urogenital sinus was reported (Rowley and Tindall, 1987; Rowley, 1992*a*). Physicochemical properties of the reported factor (UGIF) suggest that it is different from that found in the present study. However, it should be kept in mind that in both cases tests were performed on crude preparations limiting the value of these observations. Limonti *et al.* (1992) reported the antiproliferative effect of luteinising hormone-releasing hormone (LHRH) agonists on LNCaP cells. Specific binding sites were demonstrated, while receptor as well as peptide levels were thought to be negatively regulated by androgens. However, since stromal cell-derived inhibitory activity was not lost upon dialysis (cut-off 3.5 kDa), these peptides can be ruled out as candidates for the reported inhibitory activity on the basis of their molecular size. Interferons (IFNs) have also been shown to inhibit growth of prostatic epithelial cells (Deshpande *et al.*, 1989; Goldstein *et al.*, 1991; Okutani *et al.*, 1991), and, among other cell types, fibroblasts have been recognised as a rich source of interferon (arbitrarily called IFN-β) (Vilcek *et al.*, 1987). We believe, however, this is not a likely candidate either, for two reasons. First, untreated fibroblasts produce subeffective concentrations of IFN, and only treatment with 'inducers' (e.g. virus infection or double-stranded RNA chains) leads to the secretion of detectable amounts of IFN-β in the CM. Second, LNCaP cells were shown not to be inhibited by IFN-β (Goldstein *et al.*, 1991), while we demonstrated inhibition of anchorage-dependent and anchorage-independent growth of LNCaP cells by prostatic stromal cell CM.

In conclusion, the data presented in this paper demonstrate that adult prostate-derived stromal cells cultured from neoplastic lesions produce a unique factor, tentatively called 'prostate-derived epithelium inhibiting Factor' (p-EIF). On the basis of its spectrum of biological activity as well as its physicochemical and immunological properties, p-EIF can be discriminated from previously described growth inhibitors, including the TGF-β-related proteins inhibin and activin (Massague, 1987), and from interferon beta (Goldstein *et al.*, 1991). The organ-specific production and the lack of inhibition on all non-prostatic epithelial cell lines tested, emphasise the importance of this inhibitor (Kooistra *et al.*, 1995*a*). It would be very interesting to investigate whether this factor is the major 'brake' on the prostate, controlling epithelial cell proliferation and thus prostatic size. Several aspects concerning the production of p-EIF are still under investigation. Preliminary data suggest that serum-derived factors are required for optimal production and/or secretion. We failed to demonstrate significant effects of steroids (testosterone, dihydrotestosterone or oestradiol) in this respect however. Further knowledge of the factors influencing its production will facilitate purification and characterisation. Antibodies raised against the purified factor would provide us with an excellent tool for studying its role in normal physiology and its possible involvement in the development of neoplasia.

**Acknowledgements**
The authors wish to thank Dr C Knabbe (Hamburg, Germany) for stimulating discussions about this subject and Dr R Derynck (San Francisco, CA, USA) for providing us with the TGF-β1 probe. We are grateful to Dr J Trapman and Hetty van der Korput (Rotterdam, The Netherlands) for arranging TGF-β hybridisations, and Drs N Ling and F Esch for the inhibin/activin probes. In addition, we would like to thank Nancy Elissen for her excellent technical assistance regarding the neutralisation assays, Mrs Martha Bonjer for carefully proofreading the manuscript and the Foundation for Urological Research Rotterdam (SUWO) for its support.

**References**

ANTONELLI-ORLIDGE A, SAUNDERS KB, SMITH SR AND D'AMORE PA. (1989). An activated form of transforming growth factor β is produced by cocultures of endothelial cells and pericytes. *Proc. Natl Acad. Sci. USA*, **86**, 4544–4548.

BARNARD JA, LYONS RM AND MOSES HL. (1990). The cell biology of transforming growth factor beta. *Biochem. Biophys. Acta*, **1032**, 79–87.

CHEIFETZ S, LING N, GUILLEMIN R AND MASSAGUE J. (1988). A surface component on GH3 pituitary cells that recognizes transforming growth factor-beta, activin, and inhibin. *J. Biol. Chem.*, **263**, 17225–17228.

CHILDS CH. B, PROPER JA, TUCKER RF AND MOSES HL. (1982). Serum contains a platelet-derived transforming growth factor. *Proc. Natl Acad. Sci. USA*, **79**, 5312–5316.

CHOMCZYNSKI P AND SACCHI N. (1987). Single-step method of RNA isolation by acid guanidinium thiocyanate–phenol–chloroform extraction. *Anal Biochem.*, **16**, 156–159.

CHUNG LW, GLEAVE ME, HSIEH JT, HONG SJ AND ZHAU HE. (1991*a*). Reciprocal mesenchymal–epithelial interaction affecting prostate tumour growth and hormonal responsiveness. *Cancer Surv.*, **11**, 91–121.

CHUNG LWK, HONG SJ, ZHAU HE, CAMPS JL, CHANG SM, FREEMAN MR AND GAO C. (1991*b*). Fibroblast-mediated human epithelial tumor growth and hormonal responsiveness in vivo. In *Molecular and Cellular Biology of Prostate Cancer*, Karr JP, Coffey DS, Smith RG and Tindall DJ. (eds) pp. 91-100. Plenum Press: New York.

CUNHA GR, DONJACOUR AA, COOKE PS, MEE S, BIGSBY RM, HIGGINS SJ AND SUGIMURA Y. (1987). The endocrinology and developmental biology of the prostate. *Endocr. Rev.*, **8**, 338–362.

DANIELPOUR D, DART LL, FLANDERS KC, ROBERTS AB AND SPORN MB. (1989). Immunodetection and quantitation of the two forms of transforming growth factor-beta (TGF-β1 and TGF-β2) secreted by cells in culture. *J. Cell. Physiol.*, **138**, 79–86.

DERYNCK R, JARRETT JA, CHEN EY, EATON DH, BELL JR, ASSOIAN RK, ROBERTS AB, SPORN MB AND GOEDDEL DV. (1985). Human transforming growth factor-beta complementary DNA sequence and expression in normal and transformed cells. *Nature*, **316**, 701–705.

DESHPANDE N, HALLOWES RC, COX S, MITCHELL I, HAYWARD S AND TOWLER JM. (1989). Divergent effects of interferons on the growth of human benign prostatic hyperplasia cells in primary culture. *J. Urol.*, **141**, 157–160.

DJAKIEW D, DELSITE R, PFLUG B, WRATHALL J, LYNCH JH AND ONADA M. (1991). Regulation of growth by a nerve growth factor-like protein which modulates paracrine interactions between a neoplastic epithelial cell line and stromal cells of the human prostate. *Cancer Res.*, **51**, 3304–3310.

DIXON M. (1953). A nomogram for ammonium sulphate solutions. *Biochem. J.*, **54**, 457.

ESCH FS, SHIMASAKI S, COOKSEY K, MERCADO M, MASON AJ, YING SY, UENO N AND LING N. (1987). Complementary deoxyribonucleic acid (cDNA) cloning and DNA sequence analysis of rat ovarian inhibins. *Mol. Endocrinol.*, **1**, 388–396.

FEINBERG AP AND VOGELSTEIN B. (1983). A technique for radiolabeling DNA restriction endonuclease fragments to high specific activity. *Anal. Biochem.*, **132**, 6–13.

FRANKS LM, RIDDLE PN, CARBONELL AW AND GEY GO. (1970). A comparative study of the ultrastructure and lack of growth capacity of adult human prostate epithelium mechanically separated from its stroma. *J. Pathol.*, **100**, 113–119.

GLEAVE ME, HSIEH JT, VON ESCHENBACH AC AND CHUNG LW. (1992). Prostate and bone fibroblasts induce human prostate cancer growth *in vivo*: implications for bidirectional tumor-stromal cell interaction in prostate carcinoma growth and metastasis. *J. Urol.*, **147**, 1151–1159.

GOLDSTEIN D, O'LEARY M, MITCHEN J, BORDEN EC AND WILDING G. (1991). Effects of interferon βser and transforming growth factor β on prostatic cell lines. *J. Urol.*, **146**, 1173–1177.

GONZALEZ-MANCHON C AND VALE W. (1989). Activin-A, inhibin and transforming growth factor-β modulate growth of two gonadal cell lines. *Endocrinology*, **125**, 1666–1672.

GRAYCAR JL, MILLER DA, ARRICK BA, LYONS RM, MOSES HL AND DERYNCK R. (1989). Human transforming growth factor-beta 3: recombinant expression, purification, and biological activities in comparison with transforming growth factors-beta 1 and -beta 2. *Mol. Endocrinol.*, **3**, 1977–1986.

HEBERT CD AND BIRNBAUM LS. (1989). Lack of correlation between sensitivity to growth inhibition and receptor number for transforming growth factor beta in human squamous carcinoma cell lines. *Cancer Res.*, **49**, 3196–3202.

HEDGER MP, DRUMMOND AE, ROBERTSON DM, RISBRIDGER GP AND DE-KRETSER DM. (1989). Inhibin and activin regulate $^{3}$H thymidine uptake by rat thymocytes and 3T3 cells *in vitro*. *Mol. Cell. Endocrinol.*, **61**, 133–138.

HOLLEY RW, ARMOUR R AND BALDWIN JH. (1983). Activity of a kidney epithelial cell growth inhibitor on lung and mammary cells. *Cell. Biol. Int. Rep.*, **7**, 141–147.

IKEDA T, LIOUBIN MN AND MARQUARDT H. (1987). Human transforming growth factor type beta 2: production by a prostatic adenocarcinoma cell line, purification, and initial characterization. *Biochemistry*, **26**, 2406–2410.

ISAACS JT. (1984). Antagonistic effect of androgen on prostatic cell death. *Prostate*, **5**, 545–557.

KABALIN JN, PEEHL DM AND STAMEY TA. (1989). Clonal growth of human prostatic epithelial cells is stimulated by fibroblasts. *Prostate*, **14**, 251–263.

KIRD D, SZALAY MF AND KAIGHN ME. (1981). Modulation of growth of a human prostatic cancer cell line (PC-3) in agar culture by normal human lung fibroblasts. *Cancer Res.*, **41**, 1100–1103.

KNABBE C, LIPPMAN ME, WAKEFIELD LM, FLANDERS KC, KASID A, DERYNCK R AND DICKSON RB. (1987). Evidence that transforming growth factor-β is a hormonally regulated negative growth factor in human breast cancer cells. *Cell*, **48**, 417–428.

KÖNIG JJ, ROMIJN JC AND SCHRÖDER FH. (1987). Prostatic epithelium inhibiting factor (PEIF): organ specificity and production by prostatic fibroblasts. *Urol. Res.*, **15**, 145–149.

KOOISTRA A, KÖNIG JJ, ROMIJN JC AND SCHRÖDER FH. (1991). Negative control of epithelial cell proliferation by prostatic stroma. *Anticancer Res.*, **11**, 1495–1500.

KOOISTRA A, KÖNIG JJ, KEIZER DM, ROMIJN HC AND SCHRÖDER FH. (1995*a*). Inhibition of prostatic epithelial cell proliferation by a factor secreted specifically by prostatic stroma. *Prostate*, **26**, 123–132.

KOOISTRA A, ELISSEN NMJ, KÖNIG JJ, VERMEY M, VAN DER KWAST TH. H, ROMIJN HC AND SCHRÖDER FH. (1995*b*). Immunocytochemical characterization of explant cultures of human prostatic stromal cells. *Prostate* (in press).

KYPRIANOU N AND ISAACS JT. (1989). Expression of transforming growth factor-beta in the rat ventral prostate during castration-induced programmed cell death. *Mol. Endocrinol.*, **3**, 1515–1522.

LAWRENCE DA, PIRCHER R AND JULLIEN P. (1985). Conversion of a high molecular weight latent beta-TGF from chicken embryofibroblasts into a low molecular weight active beta-TGF under acidic conditions. *Biochem. Biophys. Res. Commun.*, **133**, 1026–1034.

LIMONTA P, DONDI D, MORETTI RM, MAGGI R AND MOTTA M. (1992). Antiproliferative effects of luteinizing hormone-releasing hormone agonists on the human prostatic cancer cell line LNCaP. *J. Clin. Endocrinol. Metab.*, **75**, 207–212.

McKEEHAN WL AND ADAMS PS. (1988). Heparin-binding growth factor/prostatropin attenuates inhibition of rat prostate tumor epithelial cell growth by transforming growth factor type beta. *In Vitro Cell Dev. Biol.*, **24**, 243–246.

McKEEHAN WL, ADAMS PS AND ROSSER MP. (1984). Direct mitogenic effects of insulin, epidermal growth factor, glucocorticoid, cholera toxin, unknown pituitary factors and possibly prolactin, but not androgen, on normal rat prostate epithelial cells in serum-free, primary culture. *Cancer Res.*, **44**, 1998–2010.

McNEAL JE. (1978). Origin and evolution of benign prostatic enlargement. *Invest. Urol.*, **15**, 340–345.

MARTIKAINEN P, KYPRIANOU N AND ISAACS JT. (1990). Effect of transforming growth factor-beta 1 on proliferation and death of rat prostatic cells. *Endocrinology*, **127**, 2963–2968.

MASSAGUE J. (1987). The TGF-beta family of growth and differentiation factors. *Cell*, **49**, 437–438.

MEUNIER H, RIVIER C, EVANS RM AND VALE W. (1988). Gonadal and extragonadal expression of inhibin alpha, beta A, and beta B subunits in various tissues predicts diverse functions. *Proc. Natl Acad. Sci. USA*, **85**, 247–251.

MORI H, MAKI M, OISHI K, JAYE M, IGARASHI K, YOSHIDA O AND HATANAKA M. (1990). Increased expression of genes for basic fibroblast growth factor and transforming growth factor type $\beta 2$ in human benign hyperplasia. *Prostate*, **16**, 71–80.

NISHI N, MATUO Y, NAKAMOTO T AND WADA F. (1988). Proliferation of epithelial cells derived from rat dorsolateral prostate in serum-free primary cell culture and their response to androgen. *In Vitro Cell Dev. Biol.*, **24**, 778–786.

OKUTANI T, NISHI N, KAGAWA Y, TAKASUGA H, TAKENAKA I, USUI T AND WADA F. (1991). Role of cyclic AMP and polypeptide growth regulators in growth inhibition by interferon in PC-3 cells. *Prostate*, **18**, 73–80.

PEEHL DM AND STAMEY TA. (1986). Serum-free growth of adult human prostatic epithelial cells. *In Vitro Cell Dev. Biol.*, **22**, 82–90.

ROMIJN JC, VERKOELEN CF AND SCHRÖDER FH. (1988). Application of the MTT assay to human prostate cancer cell lines *in vitro*: establishment of test conditions and assessment of hormone-stimulated growth and drug-induced cytostatic and cytotoxic effects. *Prostate*, **12**, 99–110.

ROWLEY DR AND TINDALL DJ. (1987). Responses of NBT-II bladder carcinoma cells to conditioned medium from normal urogenital sinus. *Cancer Res.*, **47**, 2955–2960.

ROWLEY DR. (1992*a*). Characterization of a fetal urogenital sinus mesenchymal cell line U4F: secretion of a negative growth regulatory activity. *In Vitro Cell. Dev. Biol.*, **28A**, 29–38.

ROWLEY DR. (1992*b*). Glucocorticoid regulation of transforming growth factor-$\beta$ activation in urogenital sinus mesenchymal cells. *Endocrinology*, **131**, 471–478.

SCHUURMANS ALG, BOLT J AND MULDER E. (1988). Androgens and TGF$\beta$ modulate the growth response to EGF in human prostate tumor cells (LNCaP). *Mol. Cell. Endocrinol.*, **60**, 101–104.

SHAH MG AND SHETH AR. (1991). Similarities in the modulation of pituitary and prostatic FSH by inhibin and related peptides. *Prostate*, **18**, 1–8.

SHERWOOD ER, FIKE WE, KOZLOWSKI JM AND LEE C. (1988). Stimulation of human epithelial cell growth by stromal cell secretory products (abstract). *Biol. Reprod.*, **38**,(Suppl.1), 86.

SHERWOOD ER, FONG CJ, LEE C AND KOZLOWSKI JM. (1992). Basic fibroblast growth factor: a potential mediator of stromal growth in the human prostate. *Endocrinology*, **130**, 2955–2963.

STORY, MT. (1991). Polypeptide modulators of prostatic growth and development. *Cancer Surv.*, **11**, 123–46.

STORY MT, LIVINGSTON B, BAETEN L, SWARTZ SJ, JACOBS SC, BEGUN FP AND LAWSON RK. (1989). Cultured human prostate-derived fibroblasts produce a factor that stimulates their growth with properties indistinguishable from b-FGF. *Prostate*, **15**, 355–365.

SUGIMURA Y, CUNHA GR AND DONJACOUR A. (1986). A morphological and histological study of castration-induced degeneration and androgen-induced regeneration in the mouse prostate. *Biol. Reprod.*, **34**, 973–983.

TENNISWOOD M. (1987). Role of epithelial-stromal interactions in the control of gene expression in the prostate: an hypothesis. *Prostate*, **9**, 375–85.

THOMPSON TC. (1990). Growth factors and oncogenes in prostate cancer. *Cancer Cells.*, **2**, 345–354.

THOMPSON TC, TRUONG LD, TIMME TL, KADMON D, McCUNE BK, FLANDERS KC, SCARDINO PT AND PARK SH. (1992). Transforming growth factor beta1 as a biomarker for prostate cancer. *J Cell Biochem.* (Suppl. 16H), 54–61.

TRUONG LD, KADMON D, McCUNE BK, FLANDERS KC, SCARDINO PT AND THOMPSON TC. (1993). Association of transforming growth factor-$\beta 1$ with prostate cancer: an immunohistochemical study. *Hum. Pathol.*, **24**, 4–9.

TUCKER RF, SHIPLEY GD, MOSES HL AND HOLLEY RW. (1984). Growth inhibitor from BSC-1 cells closely related to platelet type beta transforming growth factor. *Science*, **226**, 705–707.

VAN DEN EIJNDEN-VAN RAAIJ AJ, KOORNNEEF I, SLAGER HG, MUMMERY CL AND VAN ZOELEN EJ. (1990*a*). Characterization of polyclonal anti-peptide antibodies specific for transforming growth factor beta 2. *J. Immunol. Methods*, **133**, 107–118.

VAN DEN EIJNDEN-VAN RAAIJ AJ, VAN ZOELEN EJ, VAN NIMMEN K, KOSTER CH, SNOEK GT, DURSTON AJ AND HUYLEBROECK D. (1990*b*). Activin-like factor from *a Xenopus laevis* cell line responsible for mesoderm induction. *Nature*, **345**, 732–734.

VILCEK J, KOHASE M AND HENRIKSEN-DeSTEFANO D. (1987). Mitogenic effect of double-stranded RNA in human fibroblasts: role of autogenous interferon. *J. Cell. Physiol.*, **130**, 37–43.

WILDING G. (1991). Response of prostate cancer cells to peptide growth factors: transforming growth factor-beta. *Cancer Surv.*, **11**, 147–163.

WILDING G, ZUGMEIER G, KNABBE C, FLANDERS K AND GELMANN E. (1989). Differential effects of transforming growth factor beta on human prostate cancer cells *in vitro*. *Mol. Cell. Endocrinol.*, **62**, 79–87.